# Klebsormidin A and B, Two New UV-Sunscreen Compounds in Green Microalgal *Interfilum* and *Klebsormidium* Species (Streptophyta) From Terrestrial Habitats

**DOI:** 10.3389/fmicb.2020.00499

**Published:** 2020-03-27

**Authors:** Anja Hartmann, Karin Glaser, Andreas Holzinger, Markus Ganzera, Ulf Karsten

**Affiliations:** ^1^Department of Pharmacognosy, Institute of Pharmacy, University of Innsbruck, Innsbruck, Austria; ^2^Institute of Biological Sciences, University of Rostock, Rostock, Germany; ^3^Department of Botany, University of Innsbruck, Innsbruck, Austria

**Keywords:** mycosporine-like amino acids, terrestrial algae, UV radiation, chemotaxonomy, klebsormidin A, klebsormidin B

## Abstract

The terrestrial green algal members of the genera *Interfilum* and *Klebsormidium* (Klebsormidiophyceae, Streptophyta) are found in biological soil crusts of extreme habitats around the world where they are regularly exposed, among other abiotic stress factors, to high levels of ultraviolet radiation (UVR). As a consequence those species synthesize and accumulate either one or two mycosporine-like amino acids (MAAs), but with a missing structural elucidation up to now. Therefore, in the present study both MAAs were chemically isolated and structurally elucidated. The two new compounds exhibit absorption maxima of 324 nm. MAA 1 has a molecular weight of 467 and MAA 2 of 305, and the latter (MAA 2) was identified as N-(4,5-dihydroxy-5-(hydroxymethyl)-2-methoxy-3-oxocyclohex-1-en-1-yl)-N-methylserine using one- and two-dimensional ^1^H and ^13^C-NMR spectroscopy. MAA 1 contains an additional sugar moiety. As trivial names for these two novel MAAs we suggest klebsormidin A and klebsormidin B. Different species from all previously described phylogenetic clades of Klebsormidiophyceae were chemically screened for their MAA composition in aqueous extracts using RP−HPLC and LC−MS. The novel klebsormidin A was present throughout all clades and hence could be suitable as a chemotaxonomic marker. Additionally, controlled UVR−exposure experiments with all investigated species showed that MAA biosynthesis and intracellular enrichment is strongly induced by short wavelengths, supporting the function of these compounds as natural UV−sunscreen as well as explaining the cosmopolitan distribution and ecological success of *Interfilum* and *Klebsormidium* in terrestrial habitats.

## Introduction

Biological soil crusts (biocrusts) are regarded as lower vegetation form or cryptogamic cover, and these communities dominate drylands and disturbed ecosystems worldwide, and are well described as biodiversity and activity hotspots (Weber et al., 2016). Biocrusts are build up by living heterotrophic and phototrophic microorganisms which include bacteria, archaea, microfungi, unicellular and filamentous cyanobacteria and algae. This community can be considered as “joint venture” and “ecosystem-engineers” because as microbial activity hotspots they are important for supporting and maintaining ecosystem multifunctionality. In drylands and disturbed ecosystems biocrusts significantly contribute to primary production, biogeochemical cycling of nutrients and soil formation (Castillo-Monroy et al., 2010). These important ecological roles of biocrusts for global carbon and nitrogen cycling are reflected in about 7% C-fixation of terrestrial vegetation and 50% biological N-fixation in terrestrial habitats (Elbert et al., 2012).

The streptophyte green microalgal genera *Interfilum* and *Klebsormidium* (Klebsormidiophyceae) are key phototrophic microorganisms in biocrusts and other terrestrial habitats all over the world from polar to desert regions (Rindi et al., 2011; Karsten et al., 2014; Mikhailyuk et al., 2015; Samolov et al., 2019). Rindi et al. (2011) and Mikhailyuk et al. (2015) provided so far the most comprehensive phylogeny of both genera using various genetic markers. The latter authors introduced seven main superclades A to G, with species of the genus *Interfilum* exclusively positioned in superclade A (Mikhailyuk et al., 2008), while superclades B to G comprised all *Klebsormidium* species so far described (Rindi et al., 2011; Mikhailyuk et al., 2015). Most interesting is the observation that the assigned *Klebsormidium* lineages reflect different terrestrial habitats (Ryšánek et al., 2015), i.e., members of superclade E, for example, preferentially occur under rather humid conditions (e.g., temperate Europe). In contrast, those of superclade G are mainly found under xerophytic conditions (e.g., drylands South Africa) (Mikhailyuk et al., 2015; Samolov et al., 2019).

While aquatic phototrophic microorganisms usually do not experience desiccation and strong incident solar radiation (including ultraviolet radiation: UVR) due to the overlying water column, terrestrial *Interfilum* and *Klebsormidium* regularly face both stressors on diurnal and seasonal scales (Karsten and Holzinger, 2014). Because of anthropogenically caused stratospheric ozone loss UVR is enhanced all over the world, as well as by decreases in clouds and aerosols (Bais et al., 2015), and furthermore short wavelengths also increase with altitude (Blumenthaler, 2005). UV-A (315–400 nm) and UV-B (280–315 nm) represent a photobiological stressor for many biocrust microalgae (Karsten, 2008), since on one hand sun light is essential for photosynthesis, while on the other hand the short wavelengths of the spectrum exerts many negative effects on metabolic processes. Due to the preferential absorption of UV-B by many biomolecules, nucleic acids and proteins, for example, undergo changes in the three-dimensional molecular structure or even photo damage might happen which leads to strong disturbance of vital biochemical functions such as transcription, nucleic acid replication and translation (Buma et al., 2003; Karsten, 2008).

Since biocrust microalgae regularly experience UVR in their terrestrial habitat they depend on various physiological or metabolic strategies to mitigate or better to avoid biologically dangerous UVR effects (Karsten, 2008). Otherwise long-term survival would be at risk. The protective traits are represented by avoidance, such as growing deeper in the biocrust, thereby experiencing self-shading (Gray et al., 2007), DNA repair processes (Jones and Baxter, 2017), *de novo* biosynthesis of proteins (Karsten, 2008), as well as by the formation/accumulation of antioxidants and UV-screening compounds (Colabella et al., 2014; Rosic, 2019).

A well-studied class of organic UV-sunscreen molecules in terrestrial, freshwater and marine habitats are the so-called mycosporine-like amino acids (MAAs), which are biosynthesized and enriched by many phototrophic microorganisms and other biota (Colabella et al., 2014). MAAs are colorless, low-molecular (188–1,050 kD), water-soluble and hence polar amino acid derivatives, built up of an aminocyclo-hexenone or -hexenimine ring system which is conjugated with different substituents. The latter determine the characteristic absorbance maxima, which typically range from 310 to 360 nm in the UV-A and UV-B region (Wada et al., 2015). These organic compounds are considered to act as passive shielding biomolecules by dissipating the energy of the previously absorbed UVR as harmless heat, thereby avoiding any photochemical reaction (Bandaranayake, 1998; Fuentes-Tristan et al., 2019). The physico-chemical properties of MAAs point to pronounced high molar absorptivity for UVR as reflected in molar extinction coefficients up to 50,000. In addition, MAAs are photochemically stable molecules, and all these physico-chemical traits are prerequisites for their role as UV-sunscreen (Conde et al., 2000). While MAAs are widely studied in numerous marine organisms (Wada et al., 2015; Sun et al., 2020), much less is known for biocrust or terrestrial microalgae (Kitzing et al., 2014; Hotter et al., 2018). More recently, a chemical structure of a new MAA, called prasiolin, was reported in the terrestrial macroalga *Prasiola calophylla*, which is a member of the Chlorophyta (Trebouxiophyceae) (Hartmann et al., 2016). Prasiolin occurs among the Trebouxiophyceae only in members of the so-called *Prasiola*-clade, i.e., in *Sticchococcus*, *Trichophilus*, *Pseudomarvania*, *Prasiolopsis*, *Rosenvingiella*, etc. and hence can be used as a chemotaxonomic marker (Hotter et al., 2018). In numerous *Interfilum* and *Klebsormidium* species of the other green algal lineage Streptophyta another MAA was found (Kitzing et al., 2014; Kitzing and Karsten, 2015). Using an established HPLC approach, this streptophycean MAA exhibited a retention time of 4.7 min, which was different from prasiolin (5.5 min), although both compounds exhibited an absorbance maximum at 324 nm (Kitzing et al., 2014; Hartmann et al., 2016).

The molecular structure of this putative 324 nm-MAA in *Interfilum* and *Klebsormidium* is still not described, and hence we developed a methodological strategy to isolate, purify and elucidate the chemical structure of this new UV-sunscreen. Representative members of the seven main superclades A to G were used to clarify the qualitative distribution across lineages. Additional UV-exposure experiments with these species clearly indicate that MAA formation and accumulation can be induced by controlled UVR treatment, supporting their role as UV-sunscreen.

## Materials and Methods

### Phototrophic Microorganisms and Cultivation

Seven of the investigated *Interfilum* and *Klebsormidium* species were obtained from the Culture Collection of Algae, University of Göttingen, Germany (SAG)^1^, three additional *Klebsormidium* species were provided from the collection of the BIOTA project (Büdel et al., 2009), and three other *Klebsormidium* species were isolated from Alpine biocrusts (Mikhailyuk et al., 2015) and established as clonal cultures by the last author. Most of these samples were isolated from biological soil crusts, except *Klebsormidium elegans* (bark of oak tree) and *Klebsormidium fluitans* (freshwater lake) (Table 1). Species assignment, strain number (according to culture collections) or strain label (according to earlier publications), clade assignment of *Interfilum* and *Klebsormidium* (according to Rindi et al., 2011 and Mikhailyuk et al., 2015), and habitat information on all species studied are summarized in Table 1. The genetic identity of all species was comprehensively studied in earlier publications (Mikhailyuk et al., 2008, 2015; Rindi et al., 2011; Samolov et al., 2019).

**TABLE 1:** Species assignment, strain number (according to culture collections) or strain label (according earlier publications), genetic lineage (clade) assignment of *Interfilum* and *Klebsormidium* (according to Rindi et al., 2011; Mikhailyuk et al., 2015), habitat information on all isolates investigated.

Species	Strain number	Clade	Origin and Habitat
*Interfilum massjukiae*	SAG 2102	A	Soil on top of the surface of pyroclastic outcrops, Karadag Nature Reserve, Crimea Peninsula, Ukraine; isolated April 2005 by E. Demchenko
*Interfilum terricola*	SAG 2100	A	Soil in an oak forest, 440 m a.s.l., Haute Ardenne, Belgium; isolated 1996 by I. Kostikov
*Klebsormidium cf. flaccidium*	KUE1	B/C	Biological soil crust, 2435 m a.s.l., Limnological Station Gossenköllsee, University Innsbruck, Austria; isolated June 2009 by U. Karsten
*Klebsormidium flaccidium*	SAG 2307	B/C	Clay soil, field of beets, Niederkruechten, Lower Saxonia, Germany; isolated before 1995 by G. M. Lokhorst as strain KL 1
*Klebsormidium elegans*	SAG 7.96	D	Bark of oak tree, Staverden, The Netherlands; isolated November 1992 by G. M. Lokhorst as strain KL 24
*Klebsormidium cf. fluitans*	BOT3	E	Biocrust on concrete basement of old greenhouse, Botanical Garden, 609 m a.s.l., University of Innsbruck, Austria; isolated Apr. 2009 by U. Karsten
*Klebsormidium fluitans*	SAG 9.69	E	Lake Westeinderplassen, Rijsenhout, The Netherlands; isolated November 1992 by G. M. Lokhorst as strain KL 22
*Klebsormidium dissectum*	BOT2	E	Biocrust on concrete basement of old greenhouse, Botanical Garden, 609 m a.s.l., University of Innsbruck, Austria; isolated April 2009 by U. Karsten
*Klebsormidium nitens*	SAG 13.91	E	Tekoa soil, New Zealand; isolated 1988 by E. A. Flint
*Klebsormidium crenulatum*	SAG 2415	F	Alpine biological soil crust, 2.350 m a.s.l., Schönwieskopf next to Obergurgl, Tyrol, Austria; isolated July 2007 by A. Holzinger
*Klebsormidium deserticola*	BIOTA 14613.5e	G	Biological soil crust, 200 m a.s.l., Kuboes, Karoo Desert, South Africa, BIOTA observatory; isolated before 2006 by S. Dojani
*Klebsormidium karooense*	BIOTA 14614.18.24	G	Biological soil crust, 193 m a.s.l., Groot Derm 10, Kuboes, Namaqualand, South Africa, BIOTA observatory S21; isolated before 2006 by S. Dojani
*Klebsormidium vermiculatum*	BIOTA 14621.6	G	Biological soil crust, 35 m a.s.l., Rocherpan Nature Reserve, Piquetberg, South Africa, BIOTA observatory S29, isolated before 2006 by S. Dojani

*SAG, Collection of Algal Cultures, University of Göttingen, Germany; BIOTA, Biodiversity Monitoring Transect Analysis in Africa, German project.*

All *Interfilum* and *Klebsormidium* species were cultured on solid (1.5% agar) and in 500–1,000 mL Erlenmeyer flasks filled with liquid modified Bold’s Basal Medium (3NBBM with vitamins; Starr and Zeikus, 1993), and kept at 20°C and about 30 μmol photons m^–2^ s^–1^ under a light:dark cycle of 16:8 L:D. As light source Osram Daylight Lumilux Cool White lamps (L36W/840; Osram, Munich, Germany) were used. Radiation measurements were undertaken using a Li-Cor LI-190-SZ quantum sensor in conjunction with a Li-Cor LI-250 Light meter (LI-COR Corp., Lincoln, NE, United States). For the UVR experiments, only log-phase cultures were used.

### MAA Extraction for Purification and Isolation

**FIGURE 1:**
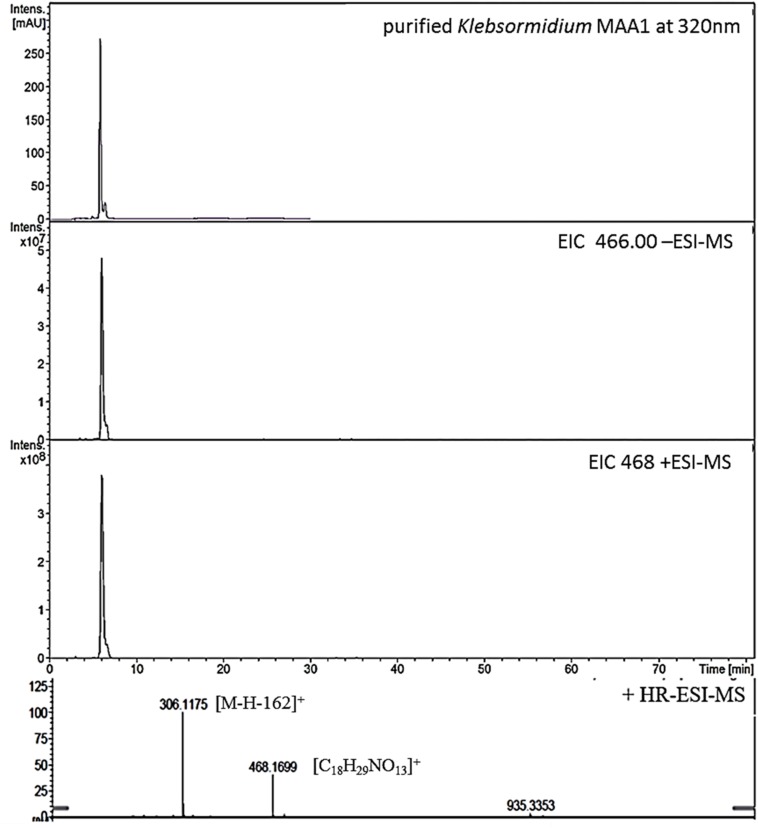
High resolution – electrospray ionization – mass spectrometry (HR-ESI-MS) spectra recorded for Compound 1 (klebsormidin A) isolated from extracts of *Klebsormidium crenulatum*.

For MAA isolation *Klebsormidium crenulatum* SAG 2415 was grown over several months under the standard conditions mentioned above which finally resulted in a biomass of 12.9 g dry weight. Such an amount is required at least to make the isolation possible. Dried material of this species was pulverized in a grinding mill followed by extraction in water/methanol (1:1) with aid of an ultrasonic bath (Bandelin Sonorex, 35 KHz) at 30°C for 30 min. Extraction was repeated twice and after centrifugation for 10 min at 5,500 × g, the supernatant was collected and evaporated at 40°C (Büchi vacuum evaporator, Switzerland). Subsequently, the dried extract (841.7 mg) was purified and fractionated on Sephadex LH 20 material using methanol/water (3:1) in a 560 × 10 mm column connected to a Büchi Crom pump B and a semi-preparative HPLC (Thermo UltiMate 3000, Waltham, MA, United States), comprising of a P580 pump, an ASI 100 automated sample injector, an UVD 170 U detector and a fraction collector. The applied gradient elution (water to methanol) resulted in 14 fractions. Fractions 7, 13, and 14 contained UV-absorbing compounds and hence were subjected to semi-preparative HPLC using a Synergi 4 μ Polar-RP column (250 × 10 mm, 4 μm; Phenomenex, Torrance, CA, United States), with a mobile phase comprising of 0.25% (v/v) formic acid in water (A) and methanol (B). The following gradient was applied: 0–8 min 2% B; 8–11 min 2–15% B; followed by re-equilibration at 2% B for 10 min; oven temperature was set to 25°C and flow rate to 2.5 mL min^–1^. Fraction 7 resulted in the isolation of MAA 1 (Figure 1). Fractions 13 and 14 were merged and resulted in the isolation of MAA 2 (Figure 3), and its chemical structure could be elucidated. MAA 1, however, was instable and degrading very fast so that an unambiguous assignment of its structure by NMR was not possible.

### Molecular Mass Determination of MAAs

To estimate the molecular mass of both new MAAs and to confirm peak purity, low-resolution mass spectra were recorded on a HP 1100 HPLC system (Agilent, Santa Clara, CA, United States), consisting of a binary pump, auto sampler, column oven and photodiode array detector. This system was coupled to an Esquire 3000 plus ion trap mass spectrometer (Bruker-Daltonics, Bremen, Germany). Separation was undertaken on a YMC-Triart C-18 column (150 × 3.0 mm; 3 μm) using a mobile phase of aqueous 20 mM ammonium acetate with 1.5% acetic acid (A) and methanol/water 80:20 with 20 mM ammonium acetate and 1.5% acetic acid (B). The flow rate was set to 0.25 mL min^–1^, the oven temperature kept at 30°C, and the injection volume was always 2 μL. The following gradient was employed: 0–5 min 2% B, 2–15% B from 5 to 15 min, 15–50% B from 15 to 20 min; from 20 to 22 min 50% B; followed by re-equilibration for 8 min at 2% B. Detection was achieved from 200 to 600 nm using specific wavelengths (310, 320, 330 nm). Mass-spectra were always recorded applying the alternating electrospray ionization (ESI) mode, keeping the temperature at 350°C, the nebulizer gas (nitrogen) at 40 psi, and a nebulizer flow (nitrogen) at 8 L min^–1^. In addition, high-resolution mass-spectra were measured by investigating the sample on a micrOTOF-Q II MS (Bruker-Daltonics, Bremen, Germany). For respective experiments the conditions were as follows: ionization in ESI mode, nebulizer gas was kept at 5.8 psi, dry gas at 4 L min^–1^ and dry temperature at 180°C. Capillary voltage was 4.0 kV (positive mode), and the scan range was between m/z 50–1,000 (Figures 1, 3).

### Structural Elucidation of MAAs

NMR experiments using the isolated compounds were conducted on a Bruker Avance II 600 and a Bruker Avance III 400 HD spectrometer (Karlsruhe, Germany) operating at 600.19 (^1^H) and 150.91 MHz (^13^C), as well as 400.13 MHz (^1^H) and 100.61 MHz (^13^C), respectively. NMR spectra were recorded at 25°C using the following experiments: ^1^H- and ^13^C- NMR, two dimensional correlation spectroscopy (2D COSY), heteronuclear multiple quantum coherence (HMQC) and heteronuclear multiple bond coherence (HMBC). All samples had to be dissolved in deuterated water, and as internal standard tetramethylsilan (both from Euriso-Top, Saint-Aubin Cedex, France) was added.^1^H and ^13^C shift values are summarized in Tables 3–5.

### UVR-Induction Experiments

For experimental verification of the induction of the new MAAs by UVR in *Interfilum* and *Klebsormidium*, always log-phase pre-cultures were used, which were grown for biomass production under the cultivation conditions mentioned above, but with a slightly enhanced photon flux density of 50 μmol photons m^–2^ s^–1^. Approximately 25 mL of these vital pre-cultures were transferred into polyacryl Petri dishes (Nümbrecht, Germany) and supplemented by 25 mL of the 3NBBM medium. As top lid of the open Petri dishes different cut-off filter foils (see below) were used to simulate two radiation scenarios for vertically incident radiation, namely PAR (photosynthetically active radiation, 400–700 nm) and PAR + UVR (PAR + UVA + UVB) treatment. A combination of radiation sources were used: daylight fluorescent tubes (Osram L 36W/954 Lumilux de lux daylight, Munich, Germany) and Q-Panel UV-A-340 fluorescent lamps (Q-Panel Company, Cleveland, OH, United States), the latter emitting an almost natural solar UV spectrum. A PMA broad-band UV radiometer (Solar Light Co., Philadelphia, PA, United States, connected to a PMA 2110 UV-A and a PMA 2106 UV-B sensor) was applied for all UVR measurements. To experimentally establish both irradiation conditions special PAR (50 μmol photons m^–2^ s^–1^) and PAR + UVR (PAR: 55 μmol photons m^–2^ s^–1^, UV-A: 8.5 W m^–2^, UV-B: 0.45 W m^–2^) cut-off filter foils were used. A 400 nm cut-off filter foil (Folex PR, Folex Dreieich, Germany) was used to exclude UVR, thereby simulating PAR conditions. For the PAR + UVR treatment a 295 nm cut-off filter foil (Ultraphan UBT, Digefra, Munich, Germany) covered the respective polyacryl Petri dishes. The MAA-induction experiments were undertaken for 4 days at 22°C under a 16:8 light–dark cycle, i.e., unchanged PAR and PAR + UVR conditions were applied during each light phase. After the treatments algal cells were harvested by filtering on pre-weighted glass fiber filters (GF6 Glasfaserfilter, Whatman GmbH, Dassel, Germany), followed by deep freezing at −70°C, freeze-drying overnight and weighting again to estimate the dried algal biomass prior MAA analysis.

### MAA Extraction and Quantification After the UVR-Induction Experiments

Freeze-dried algal cells on glass fiber filters were extracted prior HPLC analysis. Biomass on filters were transferred to screw-capped centrifuge vials filled with 5 mL 25% aqueous methanol (v/v), incubated for 2 h in a water bath at 45°C and further processed for HPLC analysis according to Karsten et al. (2009). Algal extracts were analyzed with an Agilent HPLC system (Agilent, Waldbronn, Germany), and MAAs were separated on a Phenomenex Synergi Fusion RP-18 column (4 μm, 250 × 3.0 mm I.D.) protected with a RP-18 guard cartridge (20 × 4 mm I.D.) of the same material (Phenomenex, Aschaffenburg, Germany). The mobile phase was a highly polar eluent consisting of 2.5% aqueous methanol (v/v) plus 0.1% acetic acid (v/v) in water, and adjusted to an isocratic flow rate of 0.5 mL min^–1^. The new putative MAAs were online recorded using a photodiode array detector set at 330 nm. Absorption spectra (290–400 nm) were recorded continuously in intervals of 1 s. Putative identification was done by retention time, spectral information (absorption maximum, shape) and co-chromatography with pure standards of the known MAAs palythine, porphyra-334 and shinorine. Quantification was done as total MAA concentration using the molar extinction coefficient of prasiolin (12,393 M^–1^ cm^–1^) isolated from *Prasiola calophylla* (Hartmann et al., 2016), which exhibits a similar absorption spectrum compared to both MAAs in *Interfilum* and *Klebsormidium*.

### Statistical Analysis

Always three independent replicates per treatment were used, from which the mean values and standard deviations were calculated. With the aid of one-way ANOVAs statistical significance of all mean values was analyzed. Afterward, Tukey’s *post-hoc* test was applied to record possible *a posteriori* homogeneous sub-groups of mean values that differed significantly.

## Results

### MAA Isolation and Structure Elucidation

The hydroalcoholic extract of *Klebsormidium crenulatum* was first fractionated on a Sephadex LH-20 column, and in a final purification step using semi-preparative High-Performance-Liquid Chromatography (HPLC) three UV-absorbing compounds were isolated.

#### Compound **1**

**FIGURE 2:**
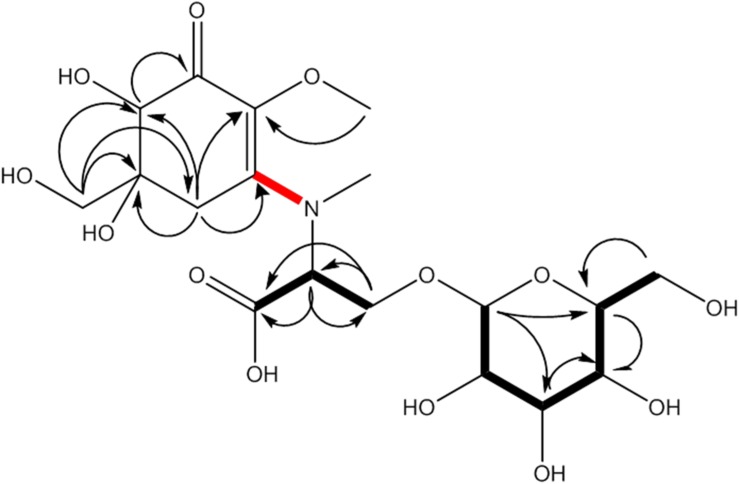
MAA compound 1 (klebsormidin A) isolated from extracts of *Klebsormidium crenulatum* was assigned to the molecular formula C_18_H_29_NO_13_, as established by a positive [M + H]^+^ ion at 468.1699. Uncertain position of the side chain due to missing long range correlation is marked in red.

This compound showed a retention time of 6.5 min, and a high resolution MS spectra was recorded ([M + H]^+^ = 468.1699; Figure 1) and compared to the calculated value ([M + H]^+^ = 468.1712) of the most likely option deduced from compound **2**. The mass deviation is <5 ppm and therefore acceptable for the tentative identification of this compound. Although this compound is instable and hence degrading fast, it was possible to record characteristic Nuclear Magnetic Resonance (NMR) shifts, which indicated the presence of a cyclohexanone-type MAA scaffold. Similar shift values for the side chain as in compound **2** pointed again to the presence of a N-methylserine, deduced from a COSY correlation of H-10/H-11 as well as one carbonyl group at 173.5. At position C-11 the side chain is additionally attached to a glucose moiety. This fact was reflected in long-range correlations visible in the HMBC spectrum (H-11a at δ_H_ 4.18 and H 11b at δ_H_ 3.94 to C-1δ_C_ 102.3), and relevant bonds between atoms are indicated by arrows in Figure 2. As no long-range correlations could be found to determine the exact location of the side chain, its position remains unclear to some extent. This is probably due to the low amount of isolated compound.

**FIGURE 3:**
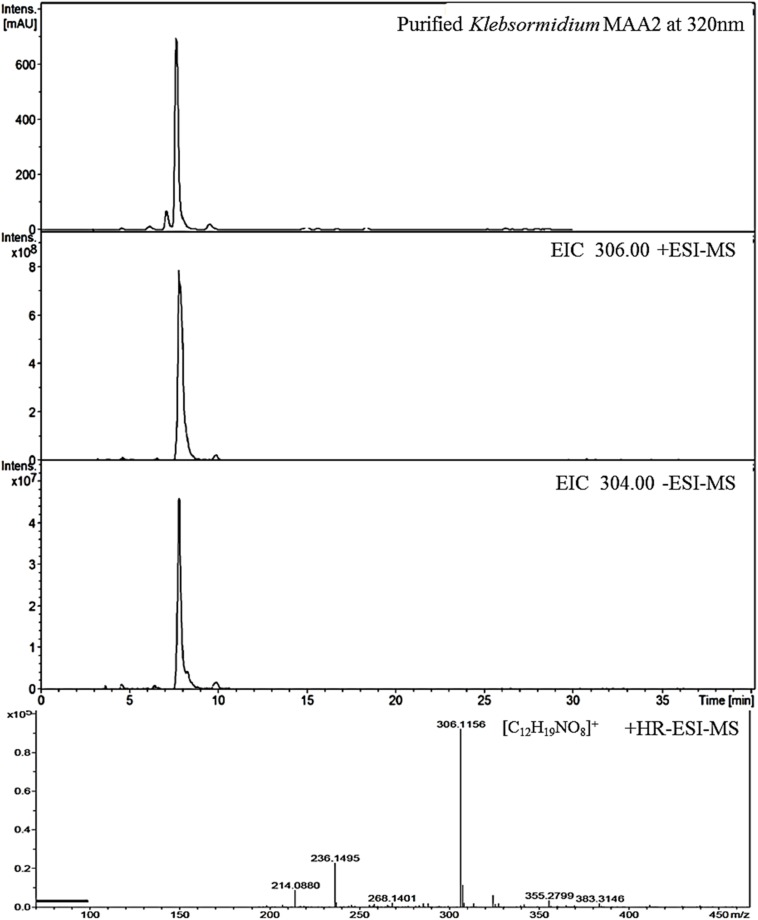
High resolution – electrospray ionization – mass spectrometry (HR-ESI-MS) spectra recorded for Compound 2 (klebsormidin B) isolated from extracts of *Klebsormidium crenulatum*.

Compound **1** was finally structurally elucidated as a new MAA, N-(4,5-dihydroxy-5-(hydroxymethyl)-2-methoxy-3-oxocyclohex-1-en-1-yl)-N-methyl-O-((2S,3R,4R,5S,6S)-3,4,5-trihydroxy-6-(hydroxymethyl)tetrahydro-2H-pyran-2-yl)-L-serinewith the molecular formulaC_18_H_29_NO_13_ (Figure 2). It was given the trivial name klebsormidin A. NMR Spectra can be found in Supplementary Figures S4A–E.

#### Compound **2**

**FIGURE 4:**
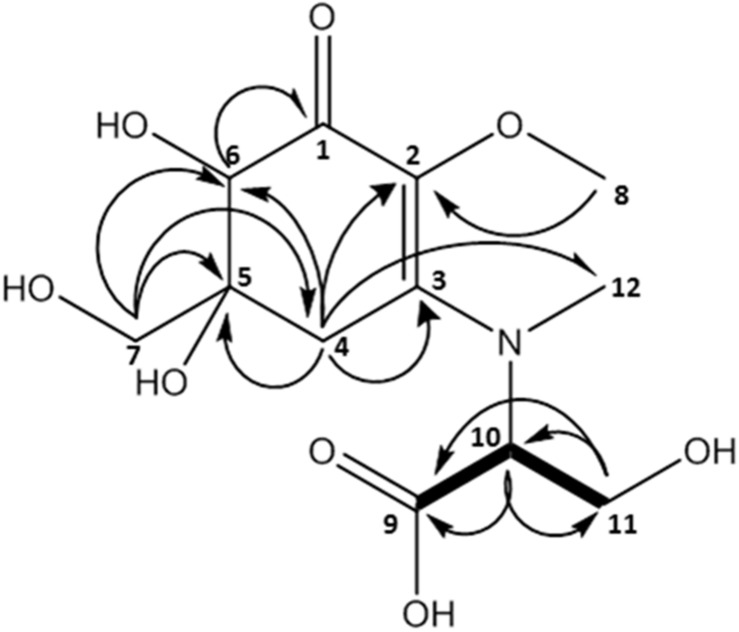
MAA compound 2 (klebsormidin B) isolated from extracts of *Klebsormidium crenulatum* was assigned to the molecular formula C_12_H_19_NO_8_ as established by a positive [M + H]^+^ ion at 306.1160.

This compound exhibited a retention time of 8.1 min, and a high resolution MS spectra was recorded ([M + H]^+^ = 306.1160; Figure 3). Characteristic NMR shifts proved the presence of a cyclohexanone-type MAA scaffold. The side chain could be again assigned to N-methylserine based on the COSY correlation of H-10/H-11 as well as on the carbonyl group at 174.6. Long-range correlations visible in the HMBC spectrum (H-4 at δ_H_ 2.88 to C-12 at δ_C_ 34.2) proved this position, and arrows point to relevant connectivities (Figure 4). Specifically, a methine group at δ_H_ 3.68 (H-10) indicated a correlation in the COSY spectrum with the protons of the methylene at δ_H_ 4.02 and δ_H_ 3.99 (H-11). The protons of the methylene at C-11 showed HMBC correlation with the carbonyl group at δ_C_ 174.6 (C-9), and those of the methyl-group at δ_H_ 2.76 (C-12) with a methine group at δ_C_ 66.8 (C-10). Compound **2** was finally structurally elucidated as a new MAA, namely N-(4,5-dihydroxy-5-(hydroxymethyl)-2-methoxy-3-oxocyclohex-1-en-1-yl)-N-methylserine, with the molecular formula C_12_H_19_NO_8_ (Figure 4). It was given the trivial name klebsormidin B.

#### Compound **3**

The ^1^H-NMR spectrum of compound **2** showed additional signals corresponding to the MAA-precursor gadusol as a degradation product. Respective shift values were comparable with literature (Grant et al., 1980) and are reported in Table 5. Respective UV chromatograms and MS spectra of the fraction containing both gadusol and klebsormidin B can be found in Supplementary Figure S1, NMR Spectra can be found in the Supplementary Figures S3A–E.

*Klebsormidium crenulatum* also contains a third MAA (absorption maxima at 322 nm, molecular weight of 275). It is only present in very low concentrations; thus it could not be isolated (Supplementary Figure S2).

Klebsormidin A and B have an absorption maximum at 324 nm, which is unusual for cyclohexanone type MAAs. Taking mycosporine-glycine as an example, they usually show the strongest absorbance at 310 nm (Rastogi and Incharoensakdi, 2014). This divergence, however, is explainable by a bathochromic effect of the additional (and unique) hydroxy group in position C 6. According to Cookson and Warijar (1956) a hydroxyl group in α-position of the ketone will result in a shift of around +17 nm, when the OH group is in an axial position. The latter is an assumption as determination of absolute stereochemistry or the prediction of UV spectra was outside the aim of this study; they will be the topic of future investigations.

### MAA Extraction and Quantification After the UVR-Induction Experiments

Representatives of the streptophyte genera *Interfilum* and *Klebsormidium* have been reported to synthesize at least one putative MAA with an absorbance maximum at 324 nm (Kitzing and Karsten, 2015). Using an improved methodological approach with a better chromatographic separation for MAAs, three peaks could be determined in the algal extracts (Table 2 and Supplementary Figure S2). While MAA 1 and MAA 2 exhibited both an absorption maximum at 324 nm, that of MAA 3 was at 322 nm. The three compounds differed in their retention time with MAA 1 at 6.5 min, MAA 2 at 8.1 min and MAA 3 at 14.0 min. The distribution patterns of these three MAAs among the investigated algal species was different. Except both E-clade representatives *Klebsormidium* cf. *fluitans* and *Klebsormidium dissectum*, which lacked MAA 3, all other species exhibited traces of this compound (Table 2). In both clade B/C members *Klebsormidium* cf. *flaccidium* and *Klebsormidium flaccidium* as well as in E-clade *Klebsormidium nitens* MAA 2 was missing. In contrast, all other *Klebsormidium* and *Interfilum* species contained at least trace amounts up to low contents of MAA 2 (Table 2). However, the quantitatively dominant UV-absorbing compound was MAA 1. Particularly, both *Interfilum* species and the F- and G-clade representatives *Klebsormidium crenulatum*, *Klebsormidium deserticola*, *Klebsormidium karooense*, and *Klebsormidium vermiculatum* exhibited the largest peaks in liquid chromatography (Table 2). The remaining taxa contained much smaller peaks.

**TABLE 2:** Species assignment, genetic lineage (clade) assignment of *Interfilum* and *Klebsormidium* (according to Rindi et al., 2011; Mikhailyuk et al., 2015), occurrence of MAAs (mycosporine-like amino acids) based on liquid chromatography and total MAA concentration (mg g^–1^ dry weight) under PAR and PAR + UVR conditions.

Species	Clade	MAA 1(324 nm)	MAA 2(324 nm)	MAA 3(322 nm)	Total MAAs under PAR (mg g^–^^1^ dw)	Total MAAs under PAR + UVR (mg g^–^^1^ dw)	Total MAAs enrichment factor
*Interfilum massjukiae*	A	XXX	X	Trace	0.89	3.77	4.2
*Interfilum terricola*	A	XXX	X	Trace	0.92	2.33	2.5
*Klebsormidium cf. flaccidium*	B/C	X	–	Trace	0.31	2.62	8.5
*Klebsormidium flaccidium*	B/C	XX	–	Trace	0.46	1.21	2.6
*Klebsormidium elegans*	D	X	X	Trace	0.34	1.74	5.1
*Klebsormidium cf. fluitans*	E	X	X	–	0.39	5.34	13.7
*Klebsormidium fluitans*	E	X	X	Trace	0.38	2.36	6.2
*Klebsormidium dissectum*	E	X	X	–	0.24	2.69	11.2
*Klebsormidium nitens*	E	Trace	–	Trace	0.18	1.62	9.0
*Klebsormidium crenulatum*	F	XXX	X	Trace	1.01	2.42	2.4
*Klebsormidium deserticola*	G	XXX	Trace	Trace	1.28	3.19	2.5
*Klebsormidium karooense*	G	XXX	X	Trace	1.65	4.16	2.5
*Klebsormidium vermiculatum*	G	XXX	X	Trace	1.42	4.08	2.9

*xxx, large peak; xx, indermediate peak; x, minor peak; –, no peak.*

**TABLE 3:** ^1^H and ^13^C NMR chemical shifts (in ppm) and proton coupling constants (Hz, in parentheses) of the novel MAA as N-(4,5-dihydroxy-5-(hydroxymethyl)-2-methoxy-3-oxocyclohex-1-en-1-yl)-N-methyl-O-((2S,3R,4R,5S,6S)-3,4,5-trihydroxy-6-(hydroxymethyl)tetrahydro-2H-pyran-2-yl)-L-serine (klebsormidin A) isolated from the terrestrial green alga *Klebsormidiumcrenulatum*.

N	^13^C	^1^H	COSY	HMBC
1	190.5	–		
2	135.5	–		
3	174.4	–		
4	40.2	a) 257, d (17.8),	4b	2,3,5,6
		b) 2.97, d (17.8)	4a	2,3,5,6
5	78.2	–		
6	75.1	4.46, s		1
7	67.7	a) 3.55, d (11.6),	7b	4,5,6
		b) 3.69, d (11.6)	7a	4,5,6
8	62.98	3.61, s		2
9	173.5	–		
10	65.52	3.85, m	11b	9
11	67.96	a) 4.18, dd (10.8, 3.0)	10, 11b	9,1
		b)3.94, m	10, 11a	9,1´
12	34.7	2.77, s		
1´	102.3	4.98, d (3.7)	2´	3´, 5´
2´	71.12	3.82, dd (10.4, 3.7)	3´, 1´	
3´	74.58	3.93, m	4´, 2´	4´
4´	64.06	3.74 d (7.6)	3´	
5´	68.14	3.93 m	6´	4´
6´	72.15	a) 3.97, m	5´	5´
		b) 3.83, m		5´

**TABLE 4:** ^1^H and ^13^C NMR chemical shifts (in ppm) and proton coupling constants (Hz, in parentheses) of the novel MAA N-(4,5-dihydroxy-5-(hydroxymethyl)-2-methoxy-3-oxocyclohex-1-en1-yl)-N-methylserine (klebsormidinB) isolated from the terrestrial green alga *Klebsormidiumcrenulatum*.

N	^13^C	^1^H	COSY	HMBC
1	192.3	–		
2	132.7	–		
3	159.2	–		
4	37.9	a) 2.99, d (17.6),	4b	3,2,6,5
		b) 2.88, d (17.6)	4a	3,12
5	76.5	–		
6	74.7	4.29, s		1
7	68.1	a) 3.62, d (11.7),	7b	4,5,6
		b) 3.55, d (11.7)	7a	4,5,6
8	63.0	3.51, s		2
9	174.6	–		
10	66.8	3.68, ov	11b	9,11
11	61.5	a) 4.02, dd (12.5, 3.6)	10, 11b	9
		b) 3.99, dd (12.5, 5.0)	10, 11a	9
12	34.2	2.76, s		10

**TABLE 5:** ^1^H and ^13^C NMR chemical shifts (in ppm) and proton coupling constants (Hz, in parentheses) of Gadusol isolated from the terrestrial green alga *Klebsormidium crenulatum*.

N	^13^C	^1^H
1	184.6	–
2	136.2	–
3	136.2	–
4	43.4	a) 2.74, dd (17.4; 2.8),
		b) 2.44, dd (17.4; 2.8)
5	78.3	–
6	75.1	4.30, s
7	68.0	a) 3.67, d (11.7),
		b) 3.56, d (11.7)
8	62.4	3.53, s

For the UV-induction experiments, first the total MAA contents in each species was determined under log-phase stock culture conditions irradiated with PAR only (see above) to gain the steady-state values (control). Afterward, these vital cultures were treated for 4 days with PAR and UVR. Under UVR-free conditions all investigated *Interfilum* and *Klebsormidium* species exhibited broad ranges in total MAA concentration from 0.18 mg g^–1^ dry weight (*K. nitens*) up to 1.65 mg g^–1^ dry weight (*K. karooense*) (Table 2). As general trend both *Interfilum* species and representatives of the F- and G-clade *Klebsormidium* exhibited 2- to 8-fold higher total MAA amounts compared to members of the B/C-, D-, and E-clade (Table 2).

All *Interfilum* and *Klebsormidium* species showed a strong induction in total MAAs after UVR exposure. While taxa with already high total MAA steady-state concentrations under PAR (e.g., G-clade) exhibited only 2–4 times increases in total MAA values after UVR treatment, the remaining species showed 5–12-fold accumulation factors and hence much stronger responses (Table 2). As a result, in all *Interfilum* and F- and G-clade *Klebsormidium* total MAA contents under UVR between 2.3 and 4.2 mg g^–1^ dry weight were measured, while in most other species this concentration ranged only between 1.6 and 2.6 mg g^–1^ dry weight. The strongest up-regulation was found in E-clade *Klebsormidium* cf. *fluitans* that exhibited the highest total MAA amount after UVR (14-fold enrichment, 5.3 mg g^–1^ dry weight) (Table 2).

## Discussion

Many members of the streptophycean lineage commonly inhabit terrestrial habitats such as soil, biological soil crusts, bark of trees, rocks or anthropogenic surfaces such as buildings, monuments, etc. (Mikhailyuk et al., 2008, 2015; Rindi et al., 2011; Ryšánek et al., 2015). From these environments in particular members of the genera *Interfilum* and *Klebsormidium* are often reported as conspicuous and abundant greenish biofilms, and hence their life-style can be characterized as atmophytic or aeroterrestrial. Consequently, these terrestrial taxa have to face strong amplitudes in the main environmental factors (Karsten and Holzinger, 2014). Although terrestrial algae have to face multiple abiotic parameters, solar radiation with strong diurnal and seasonal changes in PAR and UVR can be characterized as particularly effective (Karsten and Holzinger, 2014). The depletion of the stratospheric ozone layer is responsible for rising incident solar radiation of the UV-B range worldwide. In addition, changes in cloud cover and atmospheric aerosols, in conjunction with day length, season, latitude and altitude result in wide amplitudes in the incident solar radiation conditions of terrestrial habitats. In contrast to aquatic algae, the settlement in terrestrial habitats entails exposure to much harsher radiation conditions, in particular to UVR (Karsten and Holzinger, 2014). UVR acclimation and adaptation mechanisms are well studied and understood in marine algae and in cyanobacteria (Karsten, 2008; Gao and Garcia-Pichel, 2011 and references therein). Although about 200–250 unicellular species of aeroterrestrial green algae (Chlorophyta and Streptophyta) are morphologically described (Ettl and Gärtner, 2014), their UVR tolerance and acclimation mechanisms are much less investigated (Kitzing et al., 2014; Kitzing and Karsten, 2015; Hartmann et al., 2016).

The few available data on *Interfilum* and *Klebsormidium* indicate a generally rather broad UVR tolerance in terms of photosynthetic activity. After treatment with UVR the majority of studied taxa of *Interfilum* and *Klebsormidium* showed only a moderate drop or even no change in optimum quantum yield of photosystem II (Kitzing and Karsten, 2015), which is considered as an indicative photophysiological process for stress responses in algae (Bischof et al., 1998). Besides photosynthetic activity also growth was investigated in terrestrial green algae of the Trebouxiophyceae (Chlorophyta) under UVR exposure and only neglectable effects were found (Karsten et al., 2007). Therefore, the ecological success of terrestrial algae was explained by their typically high UVR tolerance of basic physiological processes such as photosynthesis, respiration, and growth. As fundamental underlying mechanism for such broad tolerance against UVR the biochemical capability to produce and intracellularly enrich MAAs as natural sunscreen compounds was reported (Karsten and Holzinger, 2014; Rosic, 2019). There is strong experimental evidence that MAAs indeed act like an umbrella, i.e., as passive shielding molecules which dissipate the previously absorbed UV-waveband energy as rather harmless heat, thereby avoiding the generation of any uncontrolled photochemical reaction (Rosic, 2019).

After UVR treatment all studied *Interfilum* and *Klebsormidium* species accumulated up to 14-fold higher amounts of MAAs when compared to the PAR control conditions. The highest changes were found in *Klebsormidium* clade E members, where at PAR control conditions lower amounts of MAAs were measured. These results confirm previous UVR response patterns in streptophycean algae (Karsten and Holzinger, 2014; Kitzing et al., 2014; Kitzing and Karsten, 2015). Nevertheless, it should be mentioned that the artificial UVR treatment in the laboratory was rather moderate with on average 8 W m^–2^ UV-A and 0.45 W m^–2^ UV-B. At a sunny day in late spring in Northern Germany ∼45 W m^–2^ UV-A and 1.8–2.0 W m^–2^ UV-B can be recorded (Karsten, unpublished results), and in more arid regions such as Chile or South Africa UVR will be even higher. Therefore, it is reasonable to assume that with higher UVR also more MAAs in *Interfilum* and *Klebsormidium* will be synthesized and accumulated as reported for other terrestrial green algae already (Karsten et al., 2007).

The role of MAAs as natural UV-sunscreens was studied for the first time in detail in different terrestrial cyanobacteria (Garcia-Pichel and Castenholz, 1993). These authors experimentally proved that supplemental UVR resulted in a strong MAA induction. Garcia-Pichel and Castenholz (1993) calculated that up to 26% of incident UVR was attenuated because of the presence of enriched MAAs. MAAs are still hypothesized to be intracellularly localized, and because of their high solubility they most probably occur mainly in the cytoplasm. In contrast, in the terrestrial cyanobacterium *Nostoc commune* MAAs were reported which are extracellularly anchored to the cell wall by covalent linkage to oligosaccharides of the mucilage (Ehling-Schulz et al., 1997). Due to the extracellular localization these specific MAAs attenuate even 70% of the incident UVR, thereby providing highly efficient protection against biologically harmful UV-A/B (Ehling-Schulz et al., 1997). Although klebsormidin A contains a sugar moiety which potentially could act as a chemical anchor for binding to the cell wall of *Interfilum* and *Klebsormidium*, respective data on the cellular localization of this MAA are still missing.

Besides their photo protective role, some MAAs (e.g., mycosporine-glycine) biochemically act also as antioxidant (Dunlap and Yamamoto, 1995; Kageyama and Waditee-Sirisattha, 2019). This is particularly true for the anabolic precursor of MAAs, 4-deoxygadusol (Dunlap and Shick, 1998). Therefore, the photo protective and physicochemical properties of MAAs well explain their essential function for high UV-tolerance along with antioxidant activity in algae living under harsh terrestrial environmental conditions.

Approximately 40 MAAs are known to date, and they are typically built up by a cyclohexenone or a cyclohexenimine core structure which is substituted with different amino acid moieties. The majority of them have been isolated from Rhodophyta which are described to possess the highest contents per dry weight and to contain a great range of molecule structures (Karsten, 2008).

Green algae from the chlorophytic lineage seem to comprise much lower numbers of species containing MAAs and often their molecular MAA pattern is not very complex, yet they form structurally different MAAs that are closely related to the precursor gadusol by possessing an additional hydroxyl group in position 6 of the core structure (Hartmann et al., 2016). Those compounds are found to be highly instable and it is likely that they are degrading completely during the isolation process via the intermediate gadusol. In the streptophytic lineage of green algae, evidence for the occurrence of MAAs is very scarce, and to the best of our knowledge they are totally missing in several classes including the Zygnematophyceae. In the latter, another sunscreen strategy distinct from MAAs is found, the protection by UV absorbing phenolic compounds (Remias et al., 2012; Aigner et al., 2013). Klebsormdiophyceae are early branching streptophytic green algae, with several ancient traits, and the exclusive occurrence of MAAs might be one of these.

Despite their low abundance we finally succeeded to isolate and chemically elucidate two novel MAAs from *Klebsormidium crenulatum;* N-(4,5-dihydroxy-5-(hydroxymethyl)-2-methoxy-3-oxocyclohex-1-en-1-yl)-N-methyl-O-((2S,3R,4R,5S,6S)-3,4,5-trihydroxy-6-(hydroxymethyl)tetrahydro-2H-pyran-2-yl)-L-serine which was named klebsormidin A, and N-(4,5-dihydroxy-5-(hydroxymethyl)-2-methoxy-3-oxocyclohex-1-en-1-yl)-N-methylserine which was named klebsormidin B. In addition, gadusol was identified as a degradation product. In addition, a strain of the closely related *Hormidiella attenuata* (CCAP 329-1, Klebsormidiophyceae) was also investigated in this study, but revealed a different MAA at 8.6 min with a molecular weight of 347 and absorbance maximum at 325 nm (data not shown), thus confirming earlier results (Kitzing and Karsten, 2015). In contrast to MAA-rich Rhodophyta only few chlorophytic green algae produce MAAs (Caretto and Carignan, 2011; Hartmann et al., 2016, and references therein) and even less MAAs from the streptophytic lineage are reported. As far as known, terrestrial green algae lacking MAAs or other sunscreen compounds usually avoid enhanced UVR, for example, by growing in the organic/inorganic matrix of a biofilm or biocrust, where they typically form cell aggregates or multi-layered mat-like structures which support self-shading (Karsten and Holzinger, 2014). However, many of those species still remain under-investigated, most likely due to a lack of sufficient biomass for a comprehensive chemical analysis. In addition, a full evaluation of the stability of different MAAs is experimentally still missing, and data from the literature point to the fact that there is no common physico-chemical trait which applies to all MAAs.

## Data Availability Statement

The raw data supporting the conclusions of this article will be made available by the authors, without undue reservation, to any qualified researcher.

## Author Contributions

UK and KG: ecophysiological investigation. AHa and MG: chemical analysis and isolation. AHo and KG: resources and cultivation of *Klebsormidium* and *Interfilum*. UK and AHa: writing – original draft preparation. KG, AHo, and MG: writing – review and editing. KG, AHo, MG, and UK: project administration. All authors substantially contributed to this work.

## Conflict of Interest

The authors declare that the research was conducted in the absence of any commercial or financial relationships that could be construed as a potential conflict of interest.

## Footnotes

1 www.epsag.uni-goettingen.de

## Supplementary Material

FIGURE S1 LC-MS spectra of the purified fraction containing both klebsormidin B and gadusol. Separation was carried out on a YMC-Triart C-18 column (150 × 3.0 mm; 3 μm); moblile phase **(A)** aqueous 20 mM ammonium acetate with 1.5% acetic acid and **(B)** methanol:water 90:10 with 20 mM ammonium acetate and 1.5% acetic acid and a flow of 0.25 ml min^–1^, oven was set at 30°C; injection volume 2 μL, gradient: 0–5 min 2% B, 2–15% B from 5 to 15 min, 15–50% B from 15 to 20 min, from 20 to 22 min 50% B, followed by re-equilibration for 8 min at 2% B. Detection of the peaks from 200 to 600 nm and at specific wavelengths 280, 320, 330 nm. Peak assignment: gadusol at 6.8 min, klebsormidin B at 8.1 min, MS spectra recorded in both positive and negative mode.

FIGURE S2 LC-MS spectra of the crude aqueous extract of *Klebsormidium crenulatum*. Separation was carried out on a YMC-Triart C-18 column (150 × 3.0 mm; 3μm); mobile phase **(A)** aqueous 20 mM ammonium acetate with 1.5% acetic acid and **(B)** methanol:water 90:10 with 20 mM ammonium acetate and 1.5% acetic acid and a flow of 0.25 ml min^–1^, oven was set at 30°C, injection volume 2 μL, gradient: 0–5 min 2% B, 2–15% B from 5 to 15 min, 15–50% B from 15 to 20 min, from 20 to 22 min 50% B, followed by re-equilibration for 8 min at 2% B. Detection of the peaks from 200 to 600 nm and at specific wavelengths 310, 320, 330 nm. Peak assignment based on MS data (1) klebsormidin A; (2) klebsormidin B; (4) unknown MAA. EIC spectra of unknown MAA at 14.0 min; UV spectra of unknown MAA at 14.0 min.

FIGURE S3 **(A–E)** NMR Spectra of Klebsormidin B recorded in D_2_O.

FIGURE S4 **(A–E)** NMR Spectra of Klebsormidin A recorded in D_2_O.
