# Supplementary figures and images for: Klebsormidin A and B, Two New UV-Sunscreen Compounds in Green Microalgal Interfilum and Klebsormidium Species (Streptophyta) From Terrestrial Habitats
Source: Front Microbiol. 2020 Mar 27;11:499. doi: 10.3389/fmicb.2020.00499 (PMC7118736; doi:10.3389/fmicb.2020.00499)

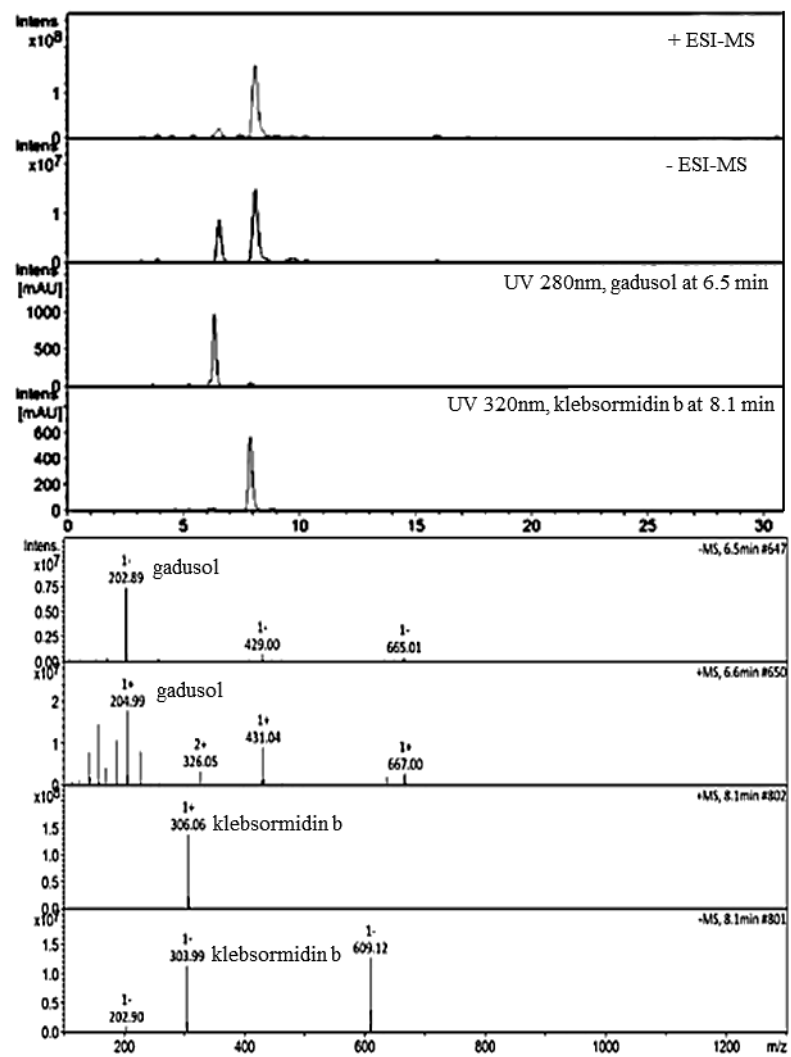

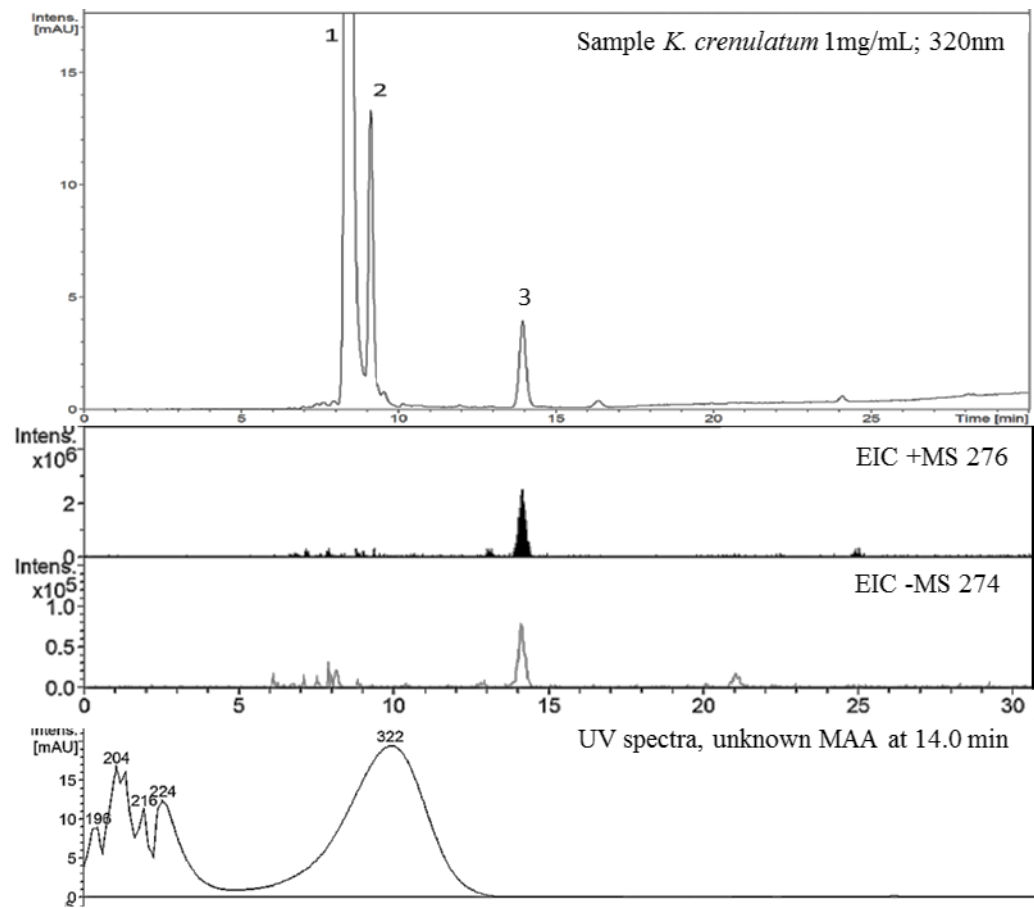

<sup>1</sup>H-NMR  
Klebs B

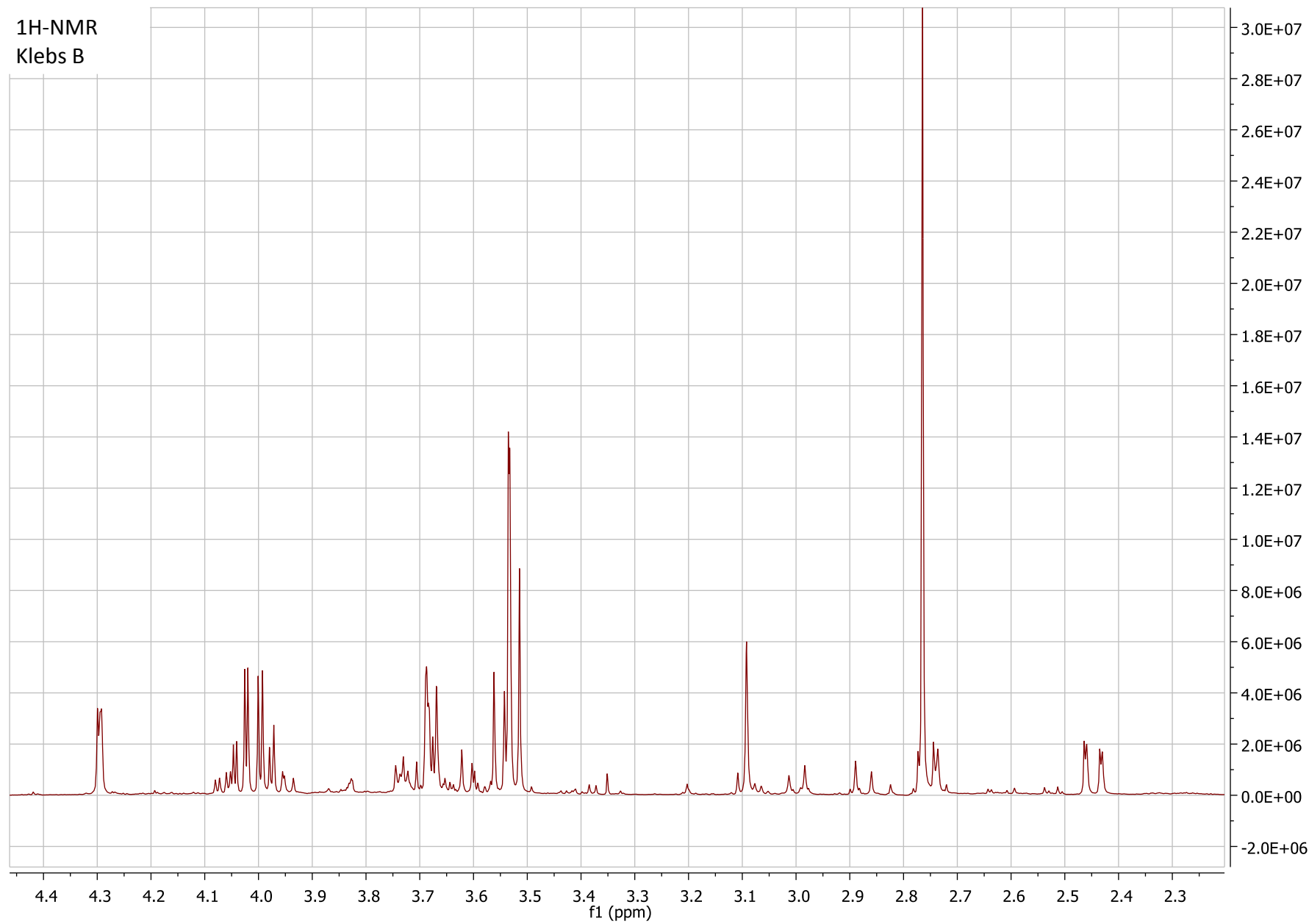

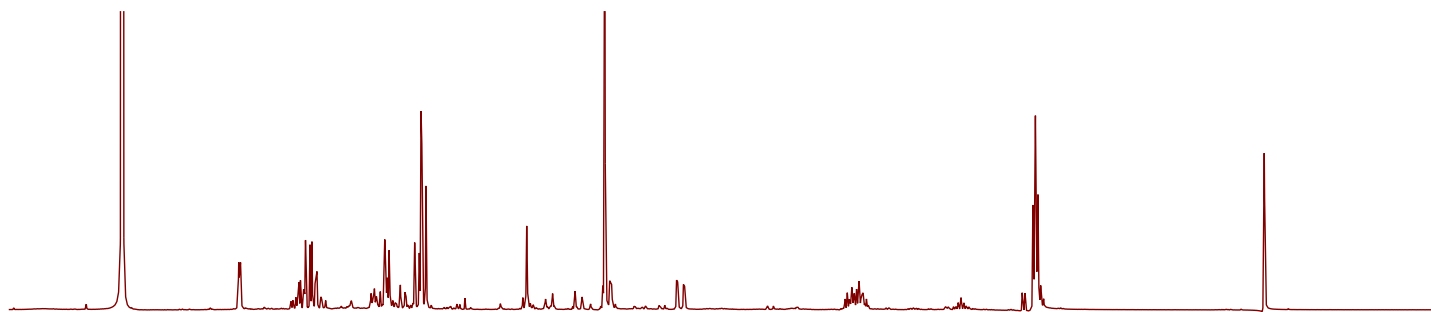

Cosy Klebs B

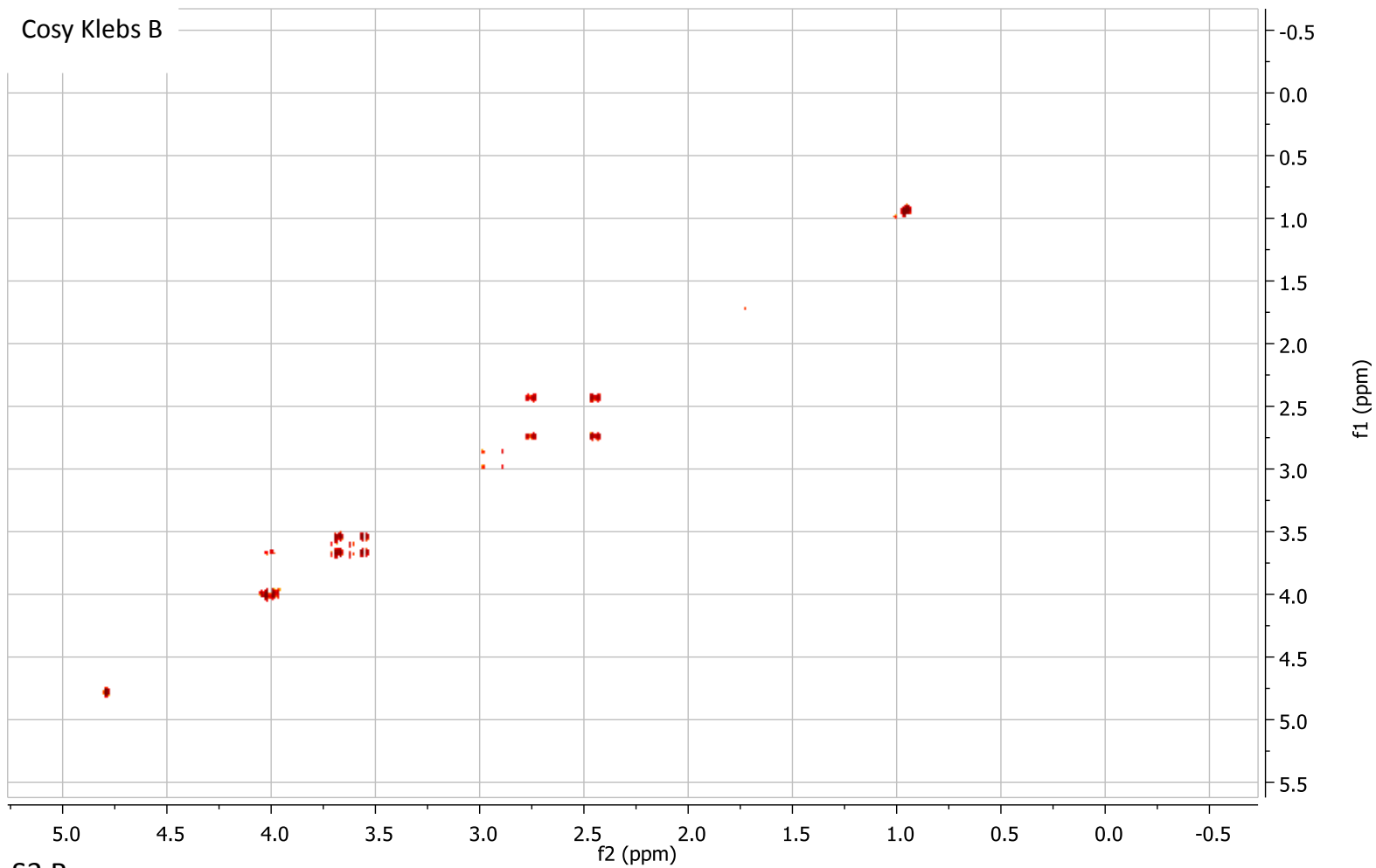

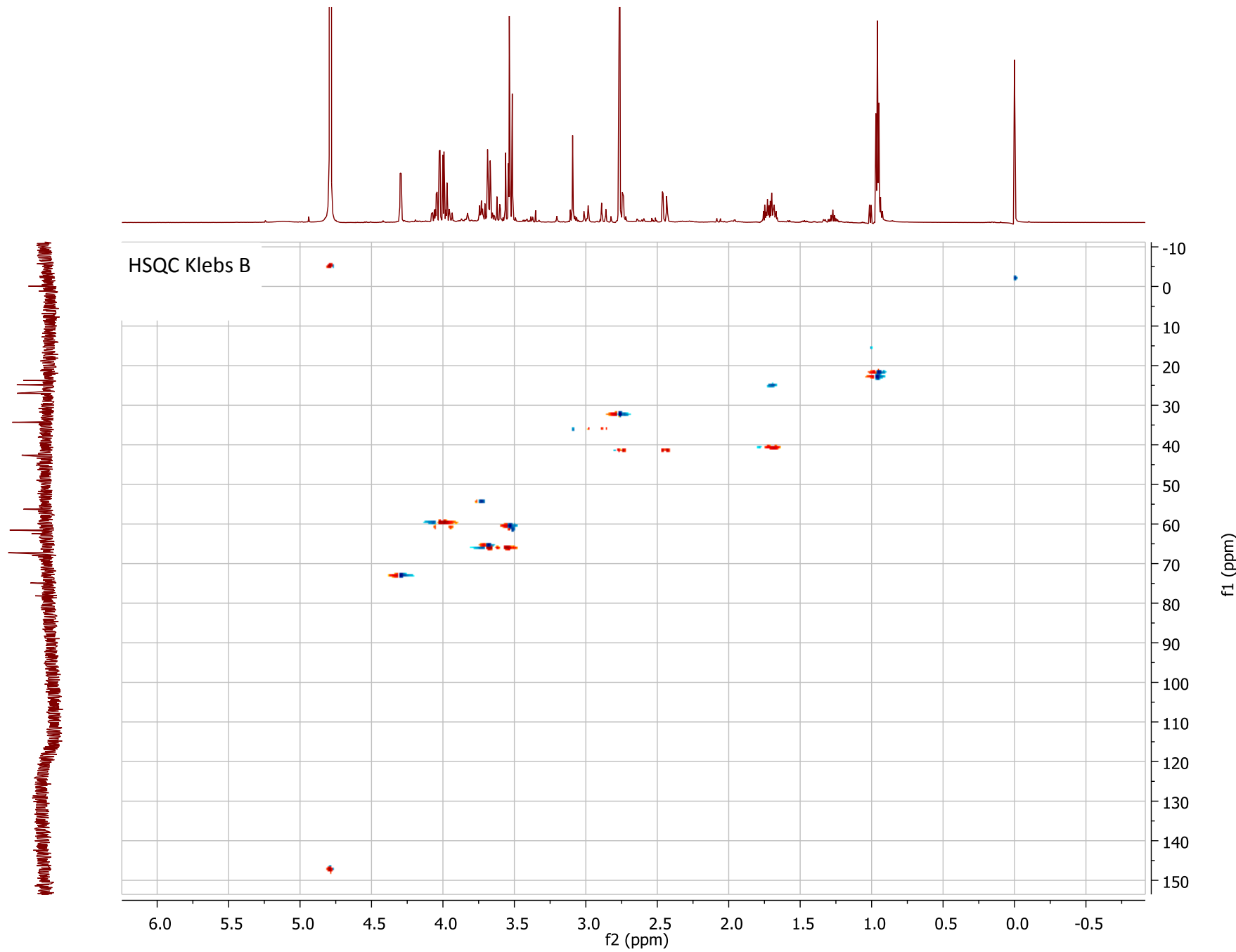

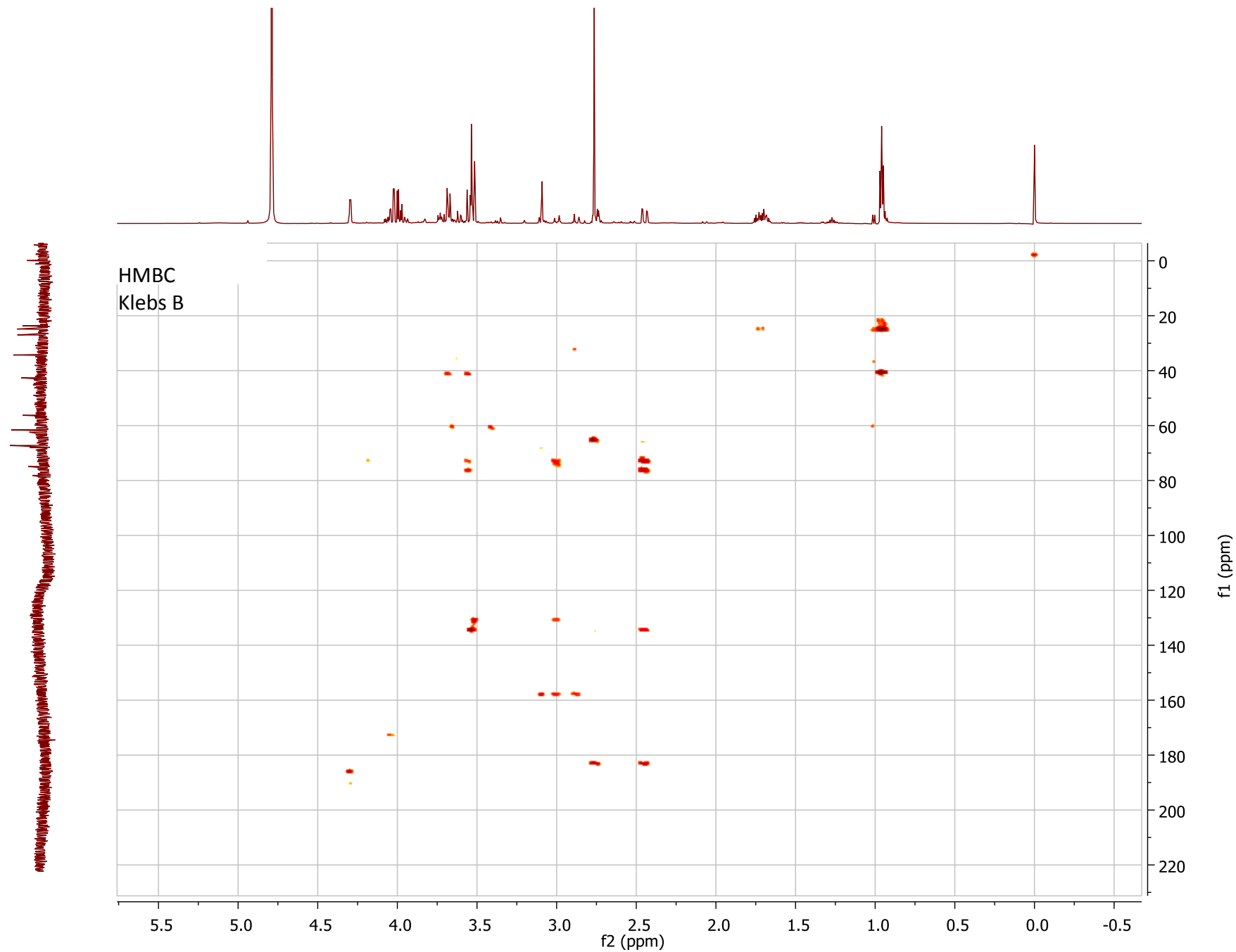

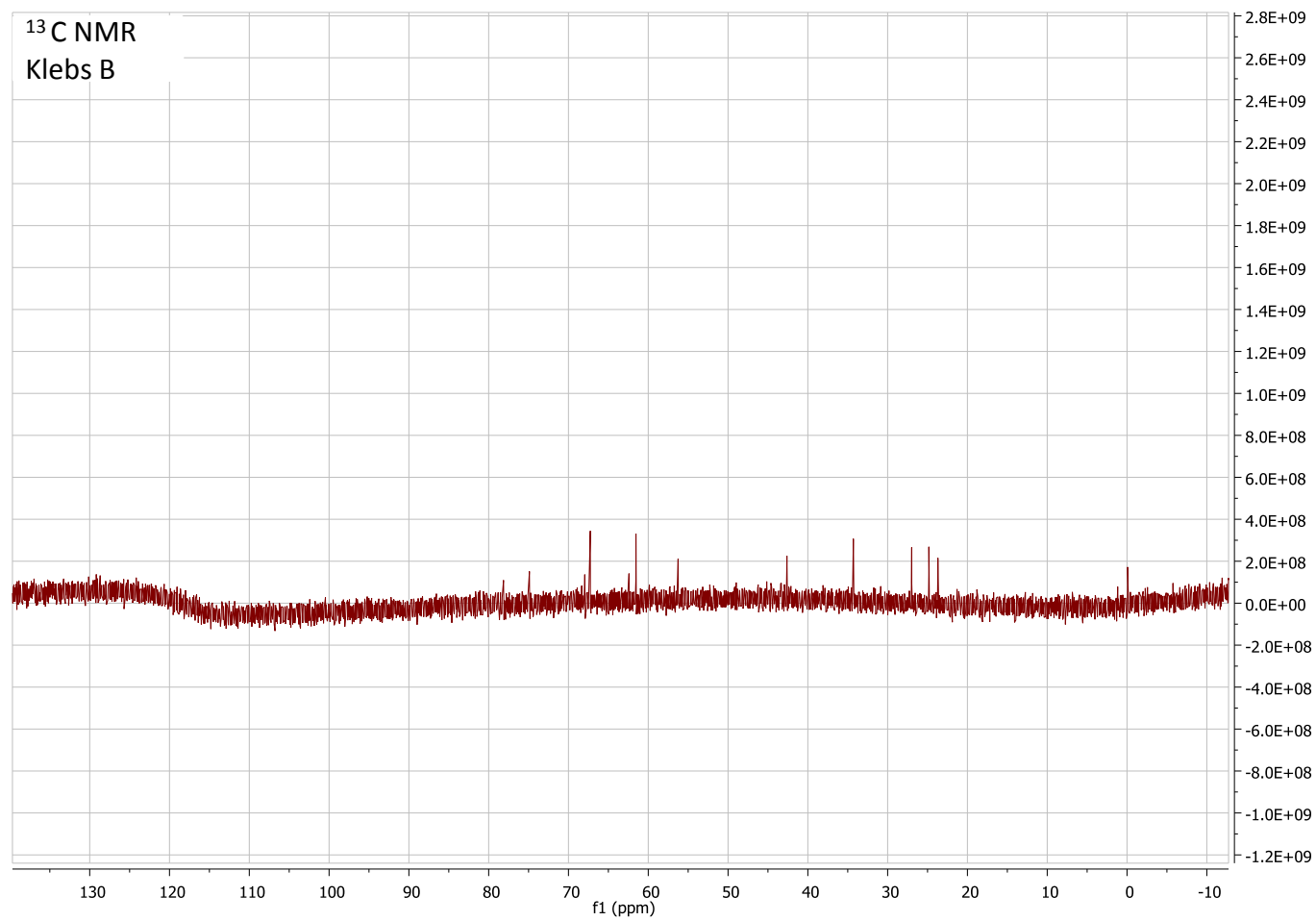

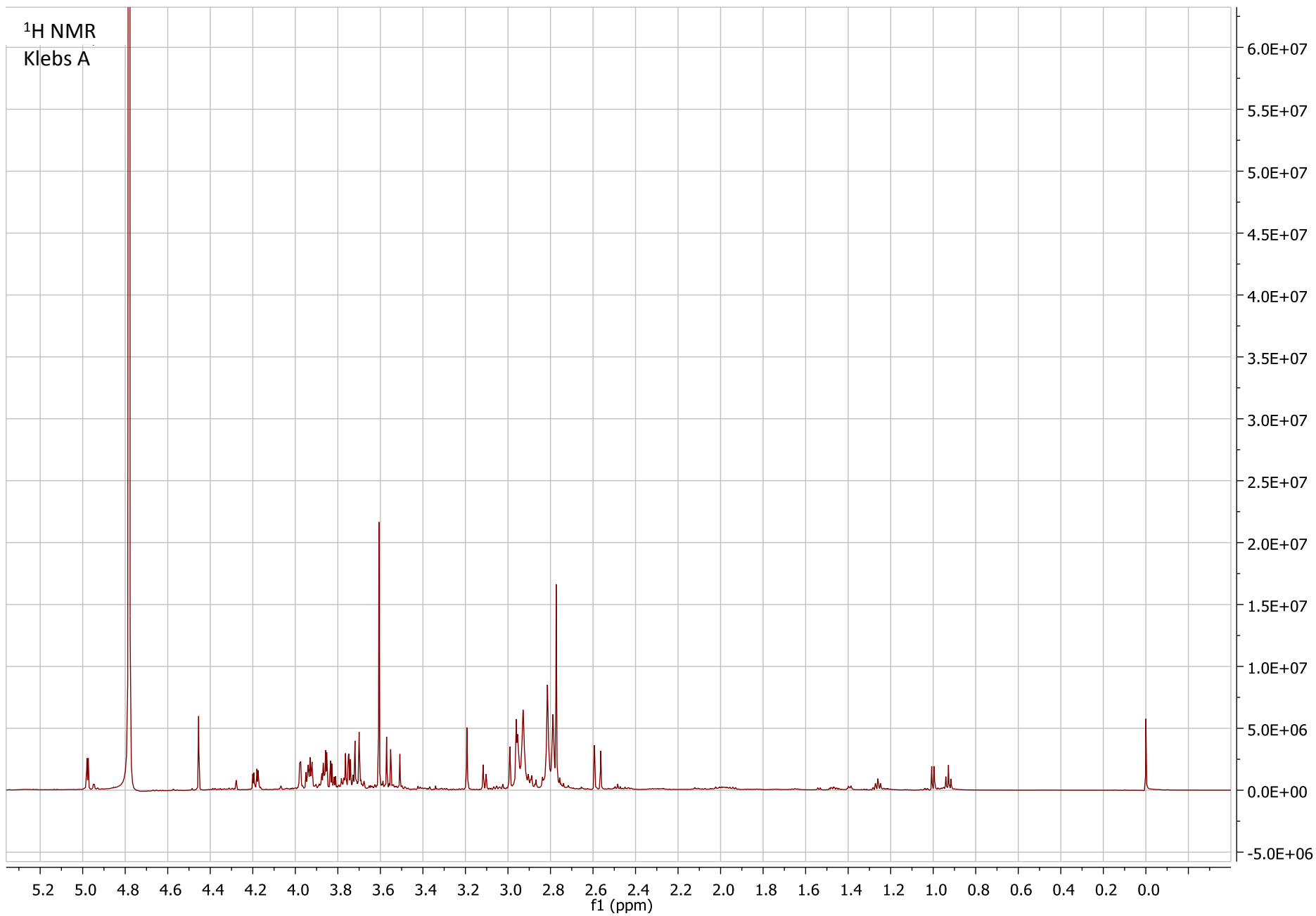

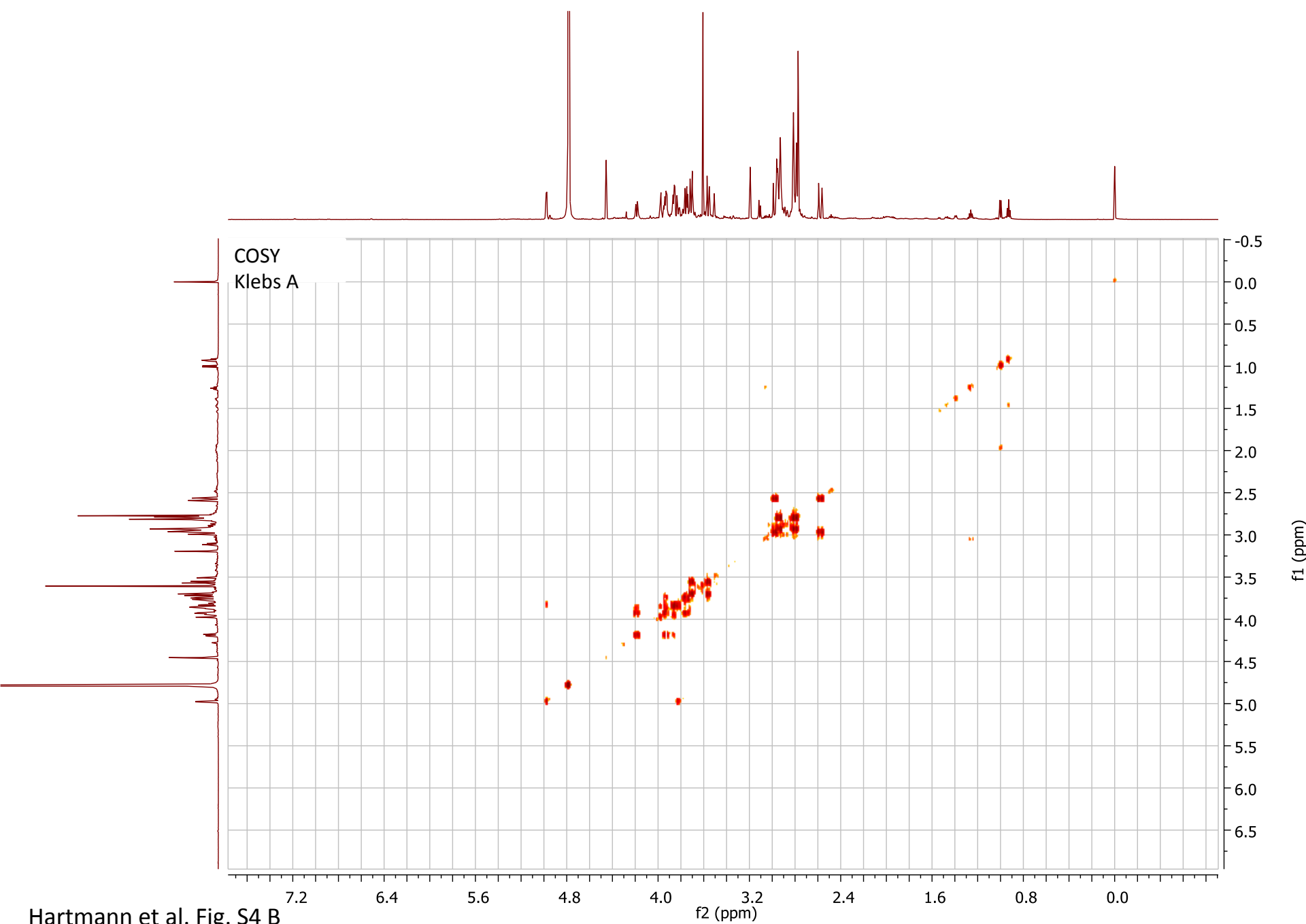

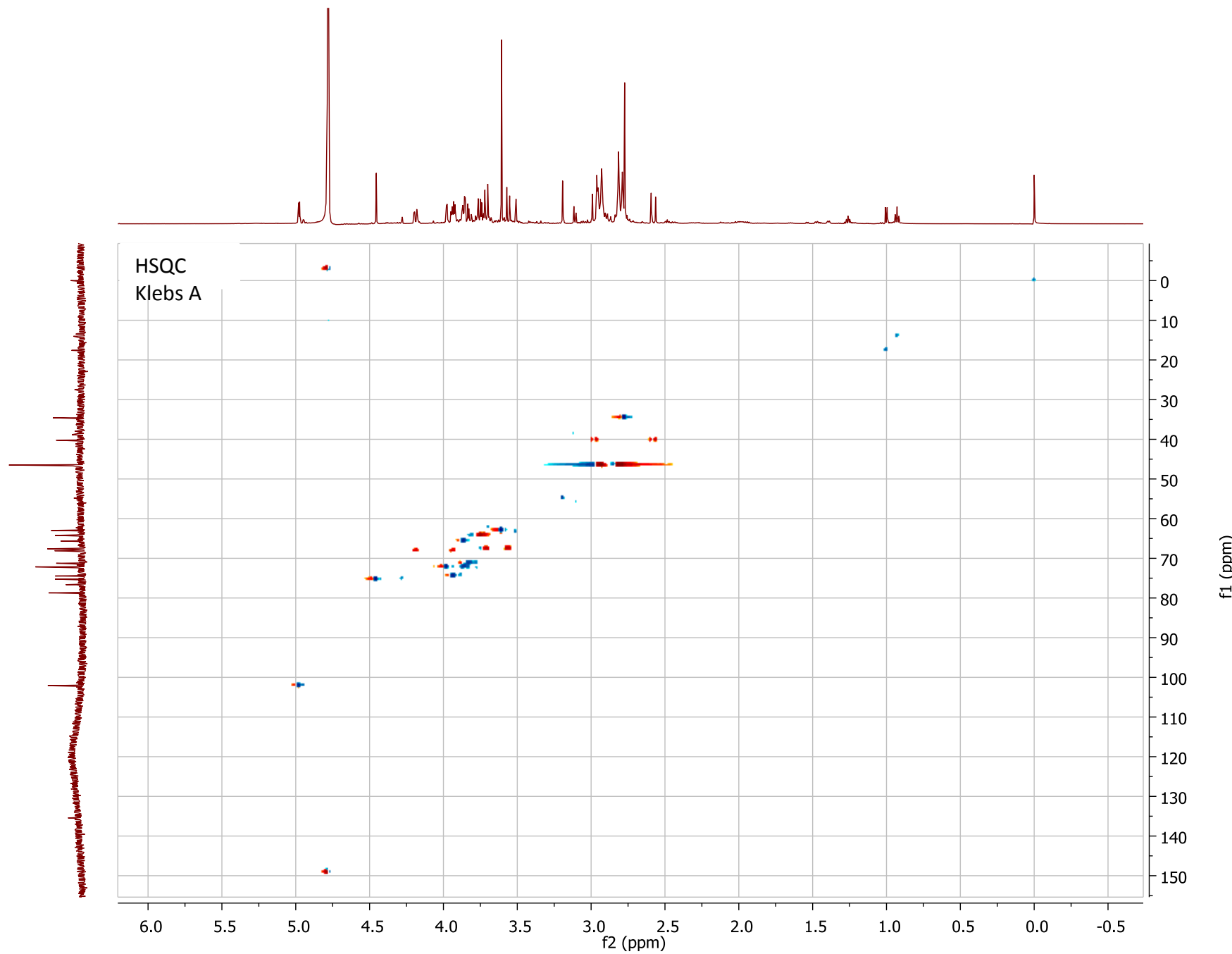

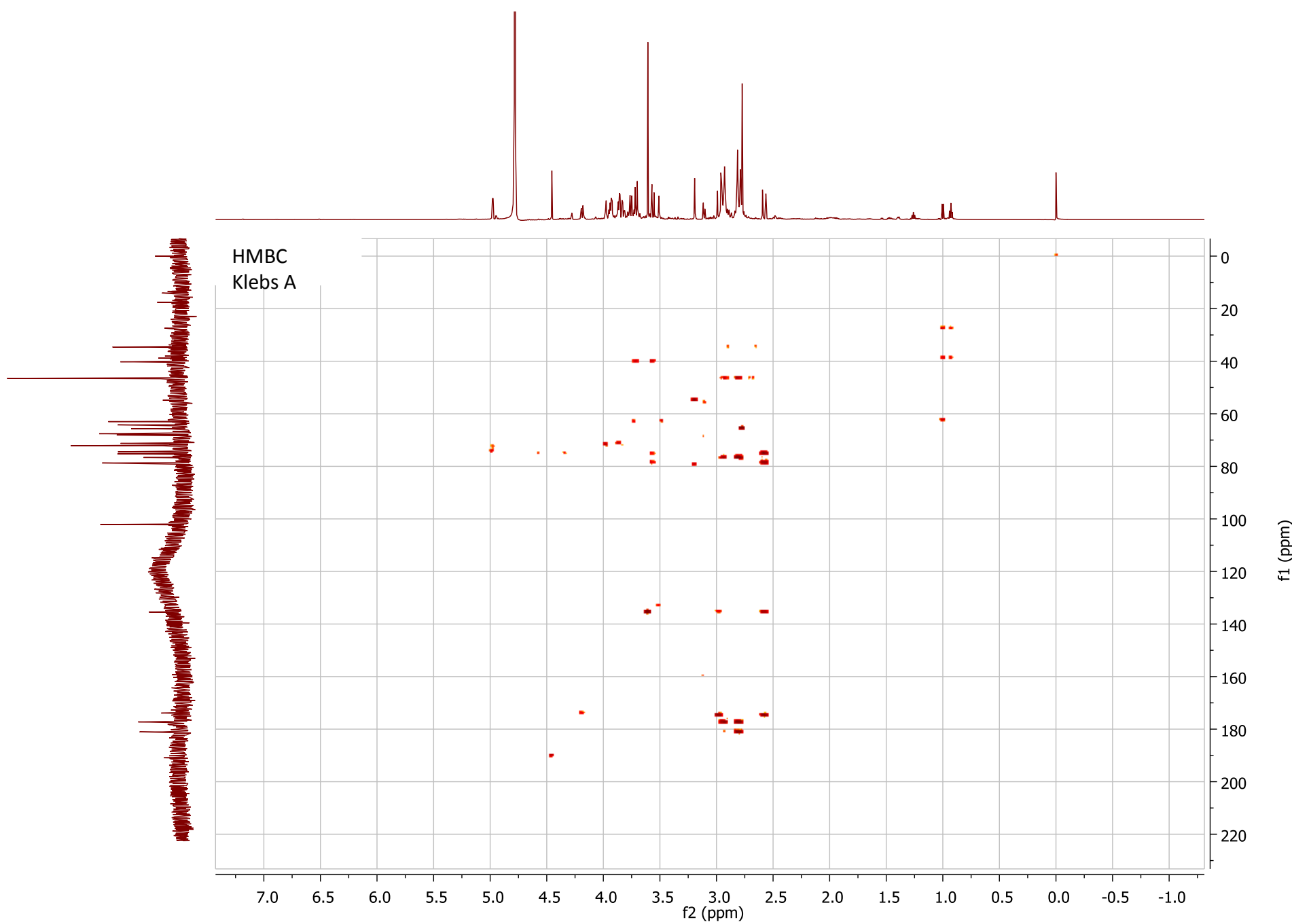

$^{13}\text{C}$  NMR  
Klebs A

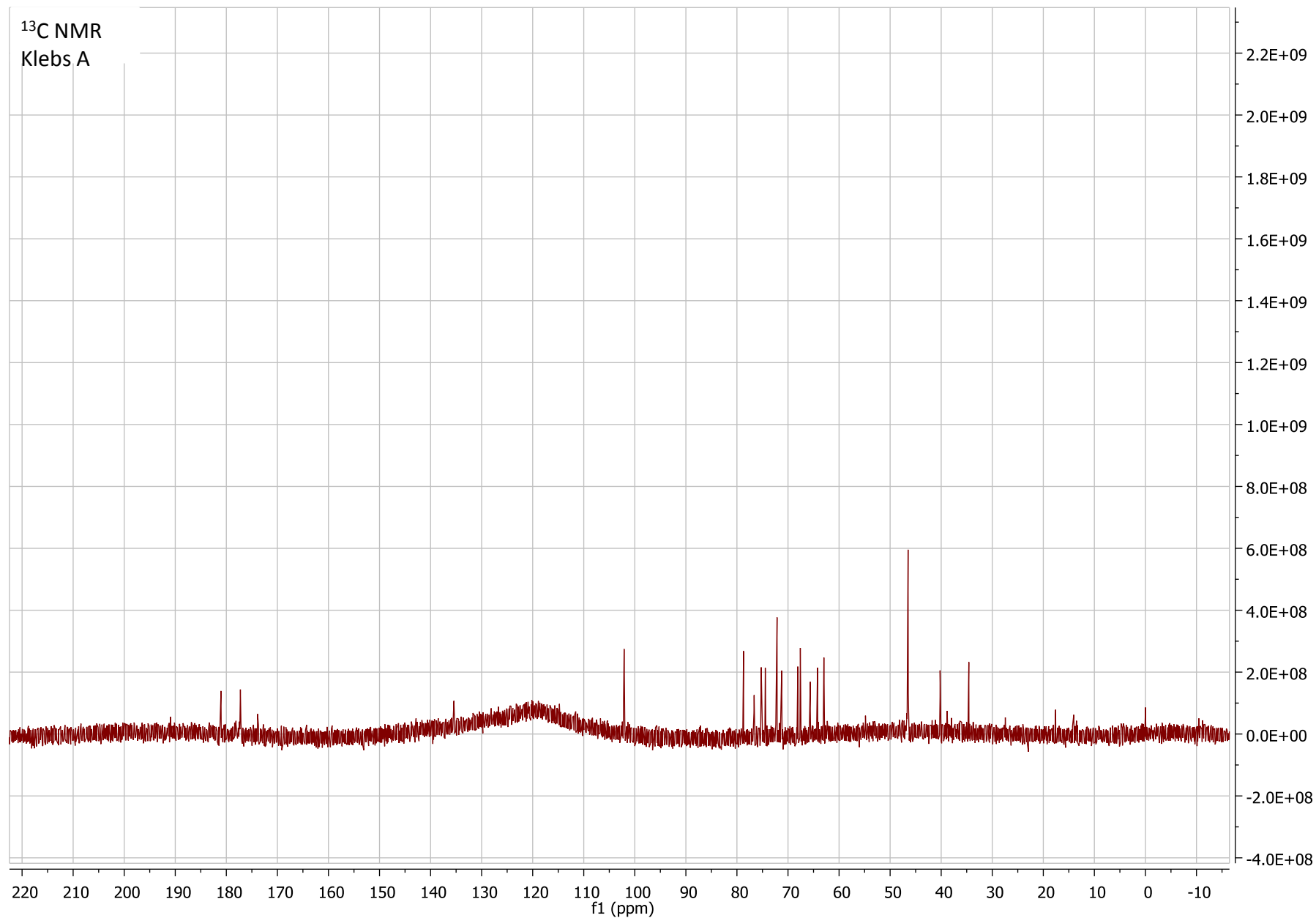

Supplement: FIGURE S1 — LC-MS spectra of the purified fraction containing both klebsormidin B and gadusol. Separation was carried out on a YMC-Triart C-18 column (150 × 3.0 mm; 3 μm); moblile phase (A) aqueous 20 mM ammonium acetate with 1.5% acetic acid and (B) methanol:water 90:10 with 20 mM ammonium acetate and 1.5% acetic acid and a flow of 0.25 ml min–1, oven was set at 30°C; injection volume 2 μL, gradient: 0–5 min 2% B, 2–15% B from 5 to 15 min, 15–50% B from 15 to 20 min, from 20 to 22 min 50% B, followed by re-equilibration for 8 min at 2% B. Detection of the peaks from 200 to 600 nm and at specific wavelengths 280, 320, 330 nm. Peak assignment: gadusol at 6.8 min, klebsormidin B at 8.1 min, MS spectra recorded in both positive and negative mode. [file Data_Sheet_1.pdf]
